# A Scoping Review of Neuromuscular Electrical Stimulation to Improve Gait in Cerebral Palsy: The Arc of Progress and Future Strategies

**DOI:** 10.3389/fneur.2019.00887

**Published:** 2019-08-21

**Authors:** Jake A. Mooney, Jessica Rose

**Affiliations:** ^1^Department of Orthopaedic Surgery, Stanford University, Stanford, CA, United States; ^2^Motion & Gait Analysis Lab, Lucile Packard Children's Hospital, Stanford Children's Health, Stanford, CA, United States

**Keywords:** NMES, FES, stimulation, cerebral palsy, gait, walking

## Abstract

**Background:** Neuromuscular deficits of children with spastic cerebral palsy (CP) limits mobility, due to muscle weakness, short muscle-tendon unit, spasticity, and impaired selective motor control. Surgical and pharmaceutical strategies have been partially effective but often cause further weakness. Neuromuscular electrical stimulation (NMES) is an evolving technology that can improve neuromuscular physiology, strength, and mobility. This review aims to identify gaps in knowledge to motivate future NMES research.

**Methods:** Research publications from 1990- July 20th 2019 that investigated gait-specific NMES in CP were reviewed using the PubMed and Google Scholar databases. Results were filtered by the National Institute of Neurological Disorder and Stroke common data elements guidelines for CP. The Oxford Centre for Evidence Based Medicine guidelines were used to determine levels of evidence for each outcome. Gait-specific NMES research protocols and trends are described, with implications for future research.

**Results:** Eighteen studies met inclusion criteria, reporting on 212 participants, 162 of whom received NMES while walking, average age of 9.8 years, GMFCS levels I–III. Studies included 4 randomized control trials, 9 cohort studies and 5 case studies. A historical trend emerged that began with experimental multi-channel NMES device development, followed by the commercial development of single-channel devices with inertial sensor-based gait event detection to facilitate ankle dorsiflexion in swing phase. This research reported strong evidence demonstrating improved ankle dorsiflexion kinematics in swing and at initial contact. Improved walking speed, step length, and muscle volume were also reported. However, improvements in global walking scores were not consistently found, motivating a recent return to investigating multi-channel gait-specific NMES applications.

**Conclusions:** Research on single-channel gait-specific NMES found that it improved ankle motion in swing but was insufficient to address more complex gait abnormalities common in CP, such as flexed-knee and stiff-knee gait. Early evidence indicates that multi-channel gait-specific NMES may improve gait patterns in CP, however significantly more research is needed. The conclusions of this review are highly limited by the low level of evidence of the studies available. This review provides a historical record of past work and a technical context, with implications for future research on gait-specific NMES to improve walking patterns and mobility in CP.

## Highlights

- Overview of gait-specific NMES for children with CP.
- Describes NMES-assisted gait technology and methods.
- Discusses history of gait-specific NMES in CP.
- Identifies gaps in knowledge and future research needs.

## Background

Cerebral palsy (CP) is the most common childhood motor disability and affects an estimated 1/323 children in the USA (1); reports of global prevalence range from 1.5 to more than 4 per 1,000 live births (2). While the initial brain injury is non-progressive, musculoskeletal impairments and functional limitations are progressive. Spastic CP affects 75% of children with CP, characterized by weak and short muscles, spasticity, and impaired selective motor control (SMC). Flexed-knee gait and stiff-knee gait are common and debilitating walking disorders in spastic CP. Many children with spastic CP lose independence in functional mobility as they age. Currently, surgical and pharmaceutical treatments for gait deficits are partially effective and often cause further muscle weakness.

Implanted or wearable electrical stimulation has been applied in a number of successful medical treatments in fields such as neurology, cardiology, and audiology. Deep brain stimulation has made groundbreaking progress in Parkinson's treatment. Electrical stimulation has been shown as an effective treatment for neurogenic bladder in patients with spinal cord injury (3) and pacemakers are now considered a standard of care in treating cardiac arrhythmias. Further, cochlear implants have returned hearing to hundreds of thousands of patients with hearing loss (4). Likewise, neuromuscular electrical stimulation (NMES) is an assistive technology in which electrical stimulation is applied either to the skin surface or via implanted electrodes to initiate or augment skeletal muscle contraction, through intact peripheral nerves (5). NMES has been used as a means to strengthen muscles in cases of stroke and CP (6), and there is promising evidence that it may do the same in cases of spinal cord injury (7).

When NMES is applied to achieve functional movements, the general term functional electrical stimulation (FES) is often used. In the context of gait, when electrical stimulation is applied during walking activities, the specific terminology used includes: gait-specific NMES or NMES-assisted gait. When stimulation is applied during functional activities related to gait such as cycling it can be termed gait-related NMES. Stimulation can also be applied as a purely muscle-strengthening application during physiotherapy, simply known as NMES. All references to electrical stimulation for the remainder of this review refer to NMES applied during walking activities, unless specified otherwise.

There is growing evidence that NMES may affect the four major neuromuscular deficits seen in spastic CP. NMES has been found to increase muscle fiber diameter and muscle size as well as strength in children with CP (8). In addition, increases in muscle fiber diameter may also increase overall muscle-tendon unit length due to the pennate angle of large lower limb muscles such as the rectus femoris and gastrocnemius. Further, there is early evidence that electrical stimulation may reduce spasticity in stroke by decreasing stretch reflex sensitivity (9, 10), indicating a need for further study of the impacts of NMES in CP. Finally, NMES may not directly improve SMC, however if applied during specific movement phases such as wrist extension when the elbow is flexed during a grasp, or knee extension when the hip is flexed at the end of swing phase of gait, it may compensate for impaired SMC, thereby improving movement patterns and functional ability.

The concept of electrical stimulation to induce muscle contractions can be traced at least as far back as 1776. The Italian physician and physicist, Luigi Galvani, famously elicited a muscular contraction from the leg of a frog using an electrical stimulus (11). Benjamin Franklin wrote about the possible role of electricity in human physiology and the digestive system in a letter to a South Carolina physician in 1757 (12). However, the successful use of NMES to achieve increased muscle strength and functional movement is a relatively new breakthrough with the majority of research conducted only in the past few decades, and on patients with spinal cord injury and stroke (6).

The miniaturization of electronics, such as inertial measurement units (IMU) and central processing units has allowed for the development of lightweight wearable NMES devices. Applied to improve gait, these devices are capable of sensing certain gait events and providing appropriate electrical stimulation in real-time (13). To date, the most common application has been single-channel stimulation to the tibialis anterior (TA) muscle during the swing phase of gait to improve foot clearance, in cases of foot drop. Control of a single muscle is termed single-channel NMES, whereas simultaneous control of multiple muscles is designated multi-channel NMES. The purpose of this review is to examine the body of literature concerning gait-specific use of single and multi-channel NMES devices to improve gait in children with CP.

## Methodology

A scoping review was chosen as the most appropriate form of review for this field at this time. The primary intention of this review was to provide a broad assessment of the historical trajectory and current state of this field of research. The secondary intention was to identify gaps in knowledge, and possible future directions. As such, a more broad-based review strategy was required. The Preferred Reporting Items for Systematic Reviews and Meta-Analyses extension for Scoping Reviews (PRISMA-ScR) Checklist was used to guide this review (14).

The literature search was conducted utilizing the PubMed database as well as Google Scholar. Additionally, relevant publications referenced through the primary search were also considered. The primary search was performed with a title and abstract keyword search using Boolean operators. Specifically, publications including “electrical stimulation” or “NMES” or “FES,” and “cerebral palsy” or “CP” in their title or abstract and published from 1990 until July 20th 2019 were collected. Only articles available in English were reviewed.

Studies were extracted that investigated gait-specific application of NMES for children with CP, reported functional classification levels of participants, and reported standard outcome metrics for gait. Specifically, the criteria of reporting common data elements (CDE) was applied. The CDE for CP are now published by the National Institute of Neurological Disorder and Stroke (NINDS) working group on CP (15) and offer validated outcome measures that can be compared between and within participants with CP. Only publications that published the Gross Motor Functional Classification (GMFCS) of participants (16) and reported at least one CDE, were included in analysis. Of note, one study published the Gillette Gait Index (GGI), a precursor to the Gait Deviation Index (GDI). Although the GGI was not explicitly endorsed by the NINDS working group, this study was included since it pre-dates the development of the GDI. The results of these publications were then assessed and levels of evidence for various outcome metrics were assigned per the Oxford Centre for Evidence -Based Medicine 2011 Level of Evidence guidelines (17).

The grading of articles and outcome measures for level of evidence was done independently by two authors (JM and JR). Articles and outcome measures were downgraded at the authors discretion for small or poorly designed studies, as allowed by the guidelines. The randomized control trials included in this review were considered level II evidence, although they were small trials.

Finally, a Cochrane risk of bias assessment for seven elements across five domains was considered and adapted to the studies being assessed (18). Since a diversity of studies, ranging from RCT to case studies were included for review, a risk of bias tool may not be an entirely appropriate assessment at this stage. However, to provide a balanced perspective on the current state of research, a summary discussion of the findings can be found in Limitations (see section Limitations).

## Results

**Figure 1:**
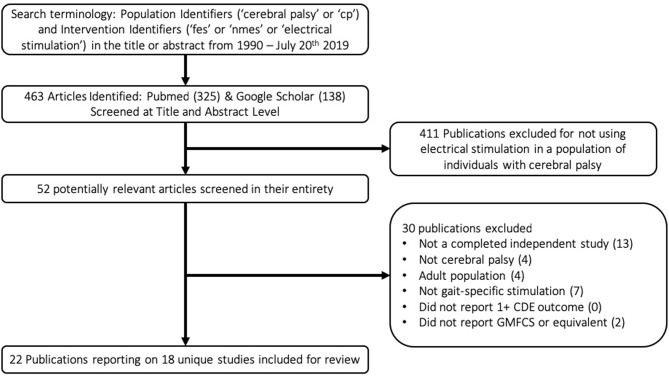
Flow chart demonstrating the scoping review process.

A flow chart schematic of the literature search and screening process can be seen in Figure 1. The search strategy outlined in the methodology resulted in 463 publications. These publications were screened at an abstract level to assess for possible relevance, specifically, if they met the broad inclusion criteria that they seemingly applied electrical stimulation in a population with CP. Studies were most commonly excluded for being done in the context of spinal cord injury or stroke, applying NMES in a non-gait-specific manner, or focusing on adult populations. This narrowed the results to 52 publications. These 52 publications were screened in their entirety to determine if they reported GMFCS levels of participants and at least one CDE outcome, in addition to confirming that they were completed studies in which NMES was used in a gait-specific manner in a pediatric population with CP. The only notable exception made was for one study (19) in which a single participant was 29 years old, and all others were under 18. The study was otherwise excellently executed with an entire year of gait-specific NMES therapy. Applying these exclusion filters eliminated 30 publications from consideration for reasons listed in the flow chart. The reasons for exclusion are listed in hierarchal order. This resulted in 22 publications reporting on 18 unique studies for inclusion in this review. Notably, the criteria of reporting at least one CDE did not solely exclude any paper.

Table 1 lists the results of the literature search, including the research study, level of evidence, as well as number, age, and functional level of participants. These 18 studies included 212 participants, 162 of whom actually received gait-specific NMES treatment, while the remainder served as controls. No study met the criteria for level 1 evidence. Four studies were small RCTs ranging from 14 to 34 total participants (level 2). Two studies were case-control cohort studies (level 3). Seven studies were cohort studies without control groups (level 3). The remaining five studies were case studies with three or fewer participants (level 4). Studies that included GMFCS I and II are most prevalent.

**Table 1:** Research studies included in the review: study design and participant demographics with study number assigned.

**Publication**	**Evidence level**	**Study design**	**Number of channels**	**Number of participants (# Control)**	**Age (*SD*)**	**GMFC*S***
1. Behbodi et al. (20)	4	Case	Multi	2 (0)	13 (0)	II–III
2. Rose et al. (21)	4	Case	Multi	3 (0)	11.3 (1.5)	I–II
3. Bailes et al. (22, 23)	3	Cohort	Single	11 (0)	9.9 (2.9)	I–II
4. Pool et al. (24–26)	2	RCT	Single	32 (16)	10.9 (3.8)	I–II
5. El-Shamy et al. (27)	2	RCT	Single	34 (17)	10.6 (0.8)	I–II
6. Khamis et al. (28)	4	Case	Single	1 (0)	18	II
7. Pool et al. (29)	3	Cohort	Single	12 (0)	9.2 (3.8)	I–II
8. Danino et al. (19)	3	Cohort	Single	4 (0)	18.5 (7.1)	I
9. Meilahn (30)	3	Cohort	Single	10 (0)	9.3 (1.7)	I
10. Prosser et al. (31); Damiano et al. (32)	3	Cohort	Single	19 (0)	12.9	I–II
11. Seifart et al. (33)	3	Cohort	Multi	5 (2)	5.1 (1.4)	I
12. Al-Abdulwahab and Al-Khatrawi (34)	2	RCT	Single	31 (10)	7.4 (2.0)	I–II^a^
13. van der Linden et al. (35)	2	RCT	Single	14 (7)^b^	8 (3.3)	I–II^c^
14. Ho et al. (36)	3	Cohort	Single	6 (0)	8.2 (2.6)	I
15. Orlin et al. (37)	3	Cohort	Multi	8 (0)	9.1 (1.3)	I–II
16. Pierce et al. (38)	4	Case	Single	1 (0)	11	I
17. Johnston et al. (39)	3	Cohort	Multi	17 (9)	7.7 (1.8)	I–III
18. Pierce et al. (40)	4	Case	Multi	2 (0)	8, 10	I

a *Estimated GMFCS determined via inclusion criteria and qualitative description of participants*.

b *All participants received some form of NMES treatment, see Table 3 for details*.

c *Gillette Functional Assessment Questionnaire was reported, estimated GMFCS is reported (41)*.

Table 2 specifies the details of the application of gait-specific NMES. The number of channels, trigger and electrode design, as well as the muscle groups targeted in each study are reported. An overall trend toward single-channel, surface stimulation to the tibialis-anterior, triggered by a tilt-sensor was observed.

**Table 2:** Research studies included in the review: NMES treatment and device design.

**Publication**	**Number of channels**	**Stimulation trigger**	**Percutaneous or surface**	**Muscle groups stimulated**
Behbodi et al. (20)	Multi (3–4)	Tilt-sensor	Surface	Glu, QF, TA, G-S^a^
Rose et al. (21)	Multi (3)	Manual	Surface	QF, Gluteus, G–S
Bailes et al. (22, 23)	Single	Footswitch	Surface	TA
Pool et al. (24–26)	Single	Tilt-sensor	Surface	TA
El-Shamy et al. (27)	Single	Tilt-sensor	Surface	TA
Khamis et al. (28)	Single	Tilt-sensor	Surface	QF
Pool et al. (29)	Single	Tilt-sensor	Surface	TA
Danino et al. (19)	Single	Footswitch	Surface	TA
Meilahn et al. (30)	Single	Tilt-sensor	Surface	TA
Prosser et al. (31); Damiano et al. (32)	Single	Tilt-sensor	Surface	TA
Seifart et al. (33)	Multi (2)	Footswitch	Surface	TA, G
Al-Abdulwahab and Al-Khatrawi (34)	Single	Continuous	Surface	GMe
van der Linden et al. (35)	Single	Footswitch	Surface	TA, QF
Ho et al. (36)	Single	Footswitch	Surface	G-S
Orlin et al. (37)	Multi (2)	Footswitch	Percutaneous	TA, G
Pierce et al. (38)	Single	Footswitch	Both	TA
Johnston et al. (39)	Multi	Footswitch	Percutaneous	TA (8), S (10), BF (2), VM (14), VL(14), PAM (2), GMe (16), GMa(16)^b^
Pierce et al. (40)	Multi (2)	Footswitch	Percutaneous	TA, G

a *Targeted muscle groups described as “ankle dorsiflexors” were assumed to be TA, and those described as “ankle plantarflexors” were assumed to be G-S*.

b *Individual participant data was not reported. Instead the cumulative number of electrodes implanted across all 8 participants was reported*.

Table 3 outlines the interventional protocol of each study, with the dosage and range of stimulator settings. If a known, commercially available NMES device was used, it is noted as well.

**Table 3:** The NMES protocol reported in each study, with dosage and stimulator settings listed to the extent reported.

**Publication**	**Intervention**	**Reported stimulator settings**
Behbodi et al. (20)	Device: Hasomed RehaStim GmbH (Maqdeburg, Germany) and custom motion sensors. Protocol: Five six-minute walking periods. NMES alternated on/off every minute within each 6-min walking bout. Achieved 60–80% maximum heart rate. Timing: Typical muscle timing and clinical recommendations. Duration: 12 weeks, 3 days/week, 30 min/day. Dosage: 1,080 min.	Frequency: 40 Hz. PW: 275–440 μs. Current: 35–60 mA.
Rose et al. (21)	Device: RT50-Z (Restorative Therapies, Baltimore, MD, USA). Protocol: Manually triggered stimulation train at IC. 30 m walk for accommodation before data collection. Timing: Typical muscle timing. Duration: Single session, 10 trials walking through the viewing volume. Dosage: <20 min.	Frequency: 40 Hz. PW: 50 μs. Current: 30 mA.
Bailes et al. (22, 23)	Device: Ness L300. Protocol: Community ambulation. Timing: Typical muscle timing. Duration: 7 days, 15 min/day (accommodation) then 12 weeks, 7 days/week, 6 h/day (intervention). Dosage: 30,345 min.	Frequency: 30–45 Hz. PW: 200–300 μs. Current: Not reported.
Pool et al. (24–26)	Device: Walkaide. Protocol: Community ambulation. Timing: Typical muscle timing. Duration: 8 weeks, 6 days/week, avg 6.2 h/day. Dosage: 17,856 min avg.	Frequency: 33 Hz. PW: 25–100 μs. Current: Variable.
El-Shamy et al. (27)	Device: Walkaide. Protocol: Community Ambulation. Timing: Typical muscle timing Duration: 7 days, 15 min/day (accommodation) then 12 weeks, at least 3 days/week, 2 h/day (intervention). Dosage: 4,425 min.	Frequency: 33 Hz. PW: 300 μs. Current: Variable.
Khamis et al. (28)	Device: Ness L300 Plus. Protocol: (1) Walking only. (2) Walking and non-gait related stimulation. (3) Walking and stair climbing 2 floors. Timing: QF stimulation from heel strike through pre-swing. Duration: (1) 20 min of accommodation. (2) 8 weeks, 7 days/week, 25 min/day of walking, 20 min of non-gait stimulation. (3) 16 weeks, 7 days/week, 30 min/day of walking and stair climbing. Dosage: 4,780 min (gait) + 1,120 min (non-gait).	Frequency: 40 Hz. PW: 300 μs. Current: 40 mA.
Pool et al. (29)	Device: Walkaide. Protocol: Community Ambulation. Timing: Typical muscle timing. Duration: 8 weeks, 6 days/week, 1 h/day. Dosage: 2,880 min.	Frequency: 25–33 Hz. PW: 25–300 μs. Current: Variable.
Danino et al. (19)	Device: Ness L300. Protocol: Community Ambulation. Timing: Typical muscle timing. Duration: 1 year, continuous daily. Dosage: Unknown.	Individually calibrated—not reported.
Meilahn (30)	Device: Walkaide. Protocol: Community Ambulation. Timing: Typical muscle timing. Duration: 12 weeks, 7 days/week, 0.9–19.5 hrs/day. Dosage: 25,724 min avg (range 6,384–55,974 min).	Frequency: Not reported. Pulse width 50 μs. Current: Not reported. Min time 0.3 sec, max time 0.6 s.
Prosser et al. (31); Damiano et al. (32)	Device: Walkaide. Protocol: Community Ambulation with (1) accommodation and (2) intervention phases. Timing: Typical muscle timing. Duration: (1) 4 weeks, 7 days/week, 30 min to 6 hrs/day. (2) 12 weeks, 7 days/week, avg 5.6 h/day (1.5–9.4 h/day). Dosage: (1) >840 min. (2) 28,224 min avg (7,560–47,376 min range).	Frequency: 16.7–33 Hz. PW: 25–300 μs. Current: Variable
Seifart et al. (33)	Device: Odstock 2 channel stimulator (O2CHSPI version 3.0, United Kingdom). Protocol: Subjects first received a botulin-toxin injection to the gastrocnemius, then home-based NMES program. Timing: Typical muscle timing. Duration: 4 weeks, 5 days/week, 30 min/day. Dosage: 600 min.	No parameters reported.
Al-Abdulwahab and Al-Khatrawi (34)	Device: Dual-channel TENS programmable stimulator model 120Z. Protocol: Laboratory based walking with (1) accommodation and (2) intervention phases. Timing: Continuous stimulation. Duration: (1) 2 minutes. (2) 7 days, 3 × 15 min/day. Dosage: 315 min.	Frequency: 20 Hz. PW: 20 μs. Current: <20 mA.
van der Linden et al. (35)	Device: Odstock. Protocol: (1) Non-gait stimulation applied at rest to either ankle DF or knee extensors in the treatment group only, followed by (2) Community ambulation. Timing: Typical muscle timing. Duration: (1) 14 days, 1 h/day. (2) 8 weeks, 4–7 days/week, continuous. Dosage: 11,520 min^a^.	Frequency: 40 Hz. PW: 3–350 μs. Current: 20–70 mA.
Ho et al. (36)	Device: Respond II Select (Medtronic Inc, Minneapolis, MN). Protocol: Laboratory-based walking. Timing: Stimulation applied from IC to toe-off. Duration: Single session, 30 trials walking through the viewing volume. Dosage: 20–30 min.	Frequency: 32 Hz. PW: 300 μs. Current: 10–40 mA.
Orlin et al. (37)	Device: Custom research device. Protocol: Laboratory-based walking, participants assigned to either TA only, gastrocnemius only, or TA and gastrocnemius. Timing: Typical muscle timing. Duration: 7 days, 2 × 45 min/day. Dosage: 630 min.	Frequency: 20–50 Hz. PW: 12–200 μs. Current: 20 mA.
Pierce et al. (38)	Device: Surface FES (S-FES) EMPI 300PV stimulator (Empi, St. Paul, MN). Percutaneous FES (P-FES) Custom research device. Protocol: Laboratory-based walking, (1) first with S-FES, (2) then P-FES. Timing: Typical muscle timing. Duration: (1) 3 months, 30 min/week. (2) 8 months, 30 min/week. Dosage: (1) 360 min S-FES. (2) 960 min P-FES.	S-FES: Frequency: 30 Hz. PW: 300 μs. Current: 20 mA. P-FES: Frequency: 20 Hz. PW: 17 μs. Current: 20 mA.
Johnston et al. (39)	Device: Custom research grade 24-channel stimulator Protocol: All study subjects underwent surgical ablative operations; the experimental group received a more limited surgery and NMES. Individualized muscle groups targeted (not reported). NMES program consisted of (1) Non-gait exercise based NMES program, then (2) gait-specific NMES. Timing: Typical muscle timing. Duration: (1) 4 weeks, 5 days/week, 1 h/day. (2) 1 year, continuous daily use. Dosage: (1) 1,200 min. (2) Unknown.	Frequency: 20 Hz. PW: up to 200 μs. Current: 20 mA.
Pierce et al. (40)	Device: Custom research device Protocol: Laboratory-based walking, each participant underwent all 3 conditions: TA only, then TA and gastrocnemius, then gastrocnemius only. Timing: Typical muscle timing. Duration: 7 days, 2 × 45 min/day. Dosage: 630 min.	Frequency: 20–50 Hz (TA) 50 Hz (gastroc.). PW: up to 200 μs. Current: 20 mA.

*PW, pulse width*.

a *From van der Linden et al. (35), “All children except one used the stimulator for 4–6 days or more a week and for 6 or more hours a day”*.

Table 4 shows the common data elements (CDE), per the NINDS guidelines, reported by each study. Improvements are compared to either baseline or a control group, as defined by each study. Carryover effects are noted. Table 5 is a report of all CDE outcomes reported by studies included in this review, organized by outcome. Relevant studies are cited, and a level of evidence for each outcome is assigned per the Oxford Center for Evidence-Based Medicine 2011 Levels of Evidence guidelines.

**Table 4:** Common data elements (CDE) outcomes reported by each study.

**Publication**	**Common data elements (CDE) reported**		
	**Improved**	**No change**	**Declined**
Behbodi et al. (20)	Step width^*^, stride length^*^, walking distance^*^, knee and ankle kinematics^*^	VO_2_, walking speed	
Rose et al. (21)	GDI	Walking speed	
Bailes et al. (22, 23)	COPM, DF at IC, 6MWT, walking speed		
Pool et al. (24–26)	Muscle volume (MRI of TA and Gastrocnemius^*^), Isometric DF strength, DF at IC, maximum ankle DF in swing, time in stance, step length, modified Tardieu^*^, COPM	SCALE, walking speed,	
El-Shamy et al. (27)	Stride length, walking speed, cadence, percent stance, VO_2_		
Khamis et al. (28)	Kinematics (maximal knee extension at midstance and at the stance phase)		
Pool et al. (29)	Ankle ROM^*^, modified Tardieu^*^, isometric DF strength^*^, concentric PF strength^*^	OGS	
Danino et al. (19)	Kinematics (ankle DF, foot progression angle), GDI		
Meilahn et al. (30)	Walking Speed	ROM (Ankle DF)	
Prosser et al. (31); Damiano et al. (32)	Kinematics (ankle DF in swing and at IC, ankle PF at TO), Muscle volume (ultrasound of TA)^*^	Walking speed, cadence, step length,	
Seifart et al. (33)	Isometric PF strength	Isometric DF strength, walking speed	
Al-Abdulwahab and Al-Khatrawi (34)	Walking speed, step length, stride length, hip adductor tone		
van der Linden et al. (35)	Ankle DF in swing and at IC, GGI		Walking speed
Ho et al. (36)		Stride length, cadence	
Orlin et al. (37)	Ankle DF in swing and at IC (TA and TA+GA only)	Walking speed, stride length	
Pierce et al. (38)	Ankle DF in swing and at IC	Stride length	Cadence, walking speed
Johnston et al. (39)	Passive ROM (hip extension/abduction, popliteal angle, knee extension, ankle DF). Temporal-spatial (step length, cadence, walking speed). GMFM (standing)	VO_2_, GMFM (crawling, walking, running, climbing)	
Pierce et al. (40)	Ankle DF in swing and at IC (TA and TA+GA only)		

*For cohort and randomized control trials, outcomes are reported as improved or declined only if reported as significant differences. For case studies, outcomes are reported as improved or declined only if ubiquitous across all subjects, otherwise reported under no change*.

* *Denotes observation of a carryover, neurotherapeutic, effect*.

**Table 5:** Common data element (CDE) outcomes across all studies, grouped by outcome metric, with study number noted.

**Outcomes**	**Improved**	**No change**	**Declined**	**Effect, level of evidence**
**TEMPORAL-SPATIAL PARAMETERS**				
Walking Speed	3, **5**, 9, **12**	2, **4**, 10, 11, 15	**13**, 16	Mixed
Step Length	**4**, **12**, 17	10		Improvement, II
Stride Length	1, **5**, **12**	14, 15, 16		Improvement, II
Step Width	1			Improvement IV
Cadence	**5**, 17	10, 14	16	Improvement, III
Time in Stance	**4**, **5**			Improvement, II
**KINEMATICS**				
**Ankle**				
DF in swing	1, **4**, 8, 10, **13**, 15, 16, 18			Improvement, I
DF at IC	1, 2, **4**, 10, **13**, 15, 16, 18			Improvement, I
PF at TO	10			Improvement, III
Foot Progression Angle	8			Improvement, IV
**Knee**				
Maximal Knee Extension	6			Improvement, IV
**PHYSIOLOGICAL OUTCOMES**				
**Muscle Volume**				
TA	**4**, 10			Improvement, II
Gastrocnemius	**4**			Improvement, II
**Muscle Strength**				
Isometric DF	**4**, 7	11		Improvement, II
Isometric PF	11			Improvement, IV
Concentric PF	7			Improvement, III
**Modified Tardieu**				
Ankle	**4**, 7			Improvement, II
**Energy Expenditure**				
VO_2_	**5**	1, 17		Improvement, II
Hip Adductor Tone (Mod. Ashworth)	**12**			Improvement, III
SCALE		**4**		No Change, II
ROM	7, 17	9		Improvement, III
**FUNCTIONAL ASSESSMENTS**				
GMFM	17	17		Improvement, III
GDI	2, 8			Improvement, IV
GGI	13			Improvement, III
COPM	3, **4**			Improvement, II
6MWT	3			Improvement, III
OGS		7		No Change, III

*Associated Oxford Centre for Evidence Based Medicine Level of Evidence is provided along with a cumulative assessment of the effect seen for a given CDE outcome metric. Randomized control trials have been bolded*.

## Discussion

### Overview

The use of electrical stimulation to improve gait in children with CP has undergone significant, and sometimes cyclic, changes over the past few decades. Early research demonstrated the approximate parameters to elicit reliable muscular contractions. This was followed by a period of exploratory NMES usage, in which a variety of NMES technologies and strategies were tested in small clinical trials. This early gait-specific usage of NMES was often ambitious, implementing complicated multi-channel systems in an attempt to normalize complex deficits. Then, driven by the development of commercial FES devices to address a single specific gait pathology in related neurological disorders, the field of NMES research in CP rapidly collapsed into the specific use of single-channel devices to augment ankle dorsiflexion in swing. This enabled a period of broader NMES usage, limited randomized controlled studies, and the first systematic reviews to be attempted. The results of this period seemed to strongly suggest that NMES can correct ankle dorsiflexion deficits in swing but is insufficient to improve more complex gait abnormalities common in CP. Now, the direction of progress is pointing back toward the use of multi-channel NMES devices.

The search criteria in this review considered publications as early as 1990, however no study met inclusion criteria until 2004. The 1990's were predominately a period of early NMES technology development in which systems capable of delivering reliable and precise electrical stimulation were developed, and the clinical applications were constrained to augmenting specific muscle strengthening exercises. It was this early body of work that allowed future investigators to make informed decisions regarding appropriate stimulation protocols. Ho et al. (36) most explicitly referenced prior work in justifying their rationalization of stimulation parameters, stating that prior work found the stimulation frequency threshold necessary to achieve fused muscle contraction (30 Hz) and the natural ramp-up time of gastrocnemius-soleus muscle contraction (0.2 s).

Standard methods and outcomes are only just emerging in this developing field. An effort was made to describe specific methods to promote standardization and repeatability. Too often, clinical expertise was cited as the rationale for customizing stimulation settings and timing. This does not promote repeatable results and limits interpretation and comparison with other studies. A concise report of stimulator settings is provided (Table 3) so that future researchers can have a better understanding of commonly used protocols. It is strongly advised to apply gait-specific NMES during normal muscle timing to promote normal sensorimotor input that can affect both neuroprosthetic and neurotherapeutic effects.

### Initial Studies: 2004–2010

NMES in CP began to be applied in a gait-specific manner and was first reported in 2004. For the first time electrical stimulation was applied synchronized with the individual's walking cycle. These early experiments were exploratory in nature, less focused on answering specific research or clinical questions, and more concerned with identifying future directions. These initial gait-specific studies and the ones that followed soon after, explored a greater diversity of potential NMES applications than has been seen since. Over this time period, 84 children participated in studies involving gait-specific NMES, 63 of whom received NMES treatment. The average age of recipients of NMES treatment during this period was 7.7 years old. All stimulation parameters were within the range of 20–50 Hz, pulse width of 3–350 μs and 10–70 mA. All studies, except one (34), applied stimulation according to normal gait timing. Percutaneous electrodes were implanted in 4 studies, and surface electrodes were used in 5, with one study directly comparing the efficacy of electrode types.

Perhaps the most defining feature of this time period in NMES usage, was the implementation of a force sensitive resistor (FSR) footswitch as the trigger for gait cycle timing and stimulation, which was ubiquitous across all studies. As a timing mechanism, this provided a reliable, albeit simplistic control scheme. Footswitches are capable of detecting initial contact and toe-off with high reliability, however, provide little to no information about the gait phase between these events. As such, this control architecture is dependent on manually preprogramming time delays into the stimulation patterns based on these two specific gait events. This approach limits real-time adaptability of the system as it is reliant on pre-determined timing, as well as its generalizability as it depends on clinician determined settings to optimize affect.

#### Single-Channel NMES-Assisted Gait

Between 2004 and 2010, four studies reported on single-channel NMES. Single-channel devices targeted the tibialis anterior (35, 38, 40), quadriceps femoris (35), gastrocnemius-soleus (36), or gluteus medius (34).

The single-channel NMES studies demonstrated that isolated stimulation of the quadriceps femoris or gastrocnemius did not significantly improve knee or ankle kinematics, respectively. Comparatively, isolated stimulation or stimulation as part of a multi-channel system of the tibialis anterior did consistently improve ankle kinematics in swing phase and at IC. One study assessing both single-channel stimulation of the tibialis anterior, and quadriceps femoris, reported significantly improved kinematics in the ankle during swing and not the knee in swing, however neither had a clinically significant impact on the GGI (35). The two studies that showed improved ankle kinematics via single-channel stimulation of the tibialis anterior also reported significantly decreased walking speeds (35, 40). The only single-channel study to demonstrate significantly improved walking speeds, stride length or step length, stimulated only the gluteus medius (34). The researchers reasoned that hip adductor spasticity was a primary cause of the stereotypical “scissor gait” seen in many patients with spastic CP, a debilitating gait. As a novel treatment strategy, they provided continual, low level stimulation to the hip abductors (gluteus medius) to promote less hip adduction. Despite the positive results of the study, continual single-channel stimulation of the gluteus medius has not been attempted since.

In all, the most persistent finding from single-channel research in this time period was that stimulation of the tibialis anterior improved ankle dorsiflexion in swing and foot clearance. Temporal-spatial parameters were significantly improved only in the study that assessed continual single-channel stimulation of the gluteus medius (34). Single-channel stimulation of either the gastrocnemius-soleus or quadriceps femoris appeared to have little effect on gait.

#### Multi-channel NMES-Assisted Gait

Between 2004 and 2010, four studies reported on multi-channel NMES. Three studies utilized two-channel devices, targeting both the gastrocnemius and tibialis anterior (33, 37, 40). One study applied NMES in a truly multi-channel manner, stimulating muscle groups actuating the hip, knee, and ankle joints (39).

All three studies that investigated ankle dorsiflexion in swing or at IC reported improvements (37, 39, 40). The results of these studies were otherwise highly variable, likely due to relatively few participants (21 children in total received NMES treatment), and inconsistent methodologies across studies. Temporal-spatial parameters were either improved or unchanged across these studies. One study assessed the effects of gait-specific NMES to the gastrocnemius and tibialis anterior after the individuals received Botox injections to the gastrocnemius and reported improved plantar flexion strength in the NMES treatment group (33).

### Recent Studies: 2010—Present

The time period since 2010 has seen significant progress in NMES technology use in the CP population. In large part, this is due to the advent of commercially available foot drop stimulators. These devices, developed initially for the stroke population, are single-channel stimulators that target the common fibular nerve to stimulate ankle dorsiflexion via the tibialis anterior in swing, thus attempting to correct foot drop, improve foot clearance, and reduce incidences of tripping. Although these devices were approved by the FDA as early as 2005, their use in pediatric populations was not approved until years later.

From 2010 to April of 2019, 128 children participated in studies involving gait-specific NMES, 99 of whom received gait-specific NMES treatment. The average age at time of treatment during this period was 10.9 years old. This is a notable increase in both number and age of participants since before 2010. The Walkaide commercial stimulator was used in 5 studies, the Bioness stimulator in 3 studies, the RT50-Z in 1 study, the Odstock 2 channel stimulator in 1 study, and the Hasomed RehaStim in 1 study. This is a drastic shift away from the research grade stimulators used in the past, to a complete uniformity in using commercial devices. Reported stimulation parameters were within the range of frequency of 16.7–45 Hz, pulse width of 25–440 μs and current of 30–60 mA. These parameters are not drastically different from the earlier studies, however notably, the use of commercial devices seemingly encouraged less precise reporting of specific parameters used. Specifically, the Walkaide and Bioness devices provide a user-controlled intensity setting, which presumably alters the current, since no study that implemented these devices in a community setting reported current settings. All studies, except one (20), applied stimulation according to normal physiological timing. All studies utilized surface electrodes. An outlier in stimulation protocols was the most recent study which utilized a multi-channel device in two individuals (20). They used a substantially higher pulse width, up to 440 μs, and current up to 60 mA. No other study reported a pulse width above 300 μs or current above 45 mA. The authors stated these pulse width and current values were determined by considering both the results of a thresholding procedure and the desired muscle activation. Further discussion explaining the deviation from prior stimulation settings was not provided. Additionally, they did not necessarily provide stimulation at normal muscle timing. As the authors explained, normal muscle timing was used as a basis, however final stimulation timing was determined by the observations and recommendations of three independent physical therapists.

Gait event detection technologies were more varied in recent studies. Two studies achieved gait event detection by a footswitch trigger, using the older model Bioness device (19, 22, 23). The newer Bioness model (28) and Walkaide studies use an inertial measurement unit (IMU) based tilt sensor. Similarly, one study (20) utilized an IMU to detect seven phases of gait by shank angular velocity. IMU based technology removes the need to install additional footswitch hardware in the sole of the user's shoe. Additionally, a tilt sensor is capable of continuous gait cycle monitoring, rather than detecting only toe-off and initial contact. This has the potential to provide a higher resolution of stimulation control, as was demonstrated in one recent study which cited the ability to detect seven phases of gait with bilateral IMUs (20). A case study of 3 children with CP, using the RT50 stimulator, had a trained observer manually trigger a stimulation chain at observation of initial contact (21).

#### Single-Channel NMES-Assisted Gait

Since 2010, most publications have applied only single-channel stimulation to the tibialis anterior, except for three case studies (Table 2). Although the focus of studies has been narrowed to largely only single-channel stimulation of the tibialis anterior, a number of significant improvements have been identified. Improvement were demonstrated in kinematics, temporal-spatial parameters, and physiological metrics such as muscle volume, muscle strength, Modified Tardieu test of spasticity, and energy expenditure. These reported improvements are likely due to a combination of delivering better NMES timing, stimulation application, and higher-powered studies. Additionally, outcome evaluations such as muscle strength and volume, were not assessed until more recently. Lasting neurotherapeutic effects have also been demonstrated. Specifically, the Modified Tardieu (26, 29) and the muscle volume of the tibialis anterior (31) and gastrocnemius (26) were reported to have significant carry-over effect, suggesting that the neuromuscular deficits of weakness and spasticity can be improved with gait-specific NMES in persons with CP.

This shift in NMES research toward a focused attention on single-channel stimulation of the tibialis anterior to augment dorsiflexion brought both benefits and potential missed opportunities. Such ubiquitous attention has allowed for the first credible systematic review to be attempted in the field (8). With a review question limited to the effect of functional electrical stimulation during walking on ankle dorsiflexors in children with CP, the reviewers concluded that improvements in active ankle dorsiflexion range of motion, strength, selective motor control, balance and gait kinematics could be achieved. However, they could not draw a conclusion on whether or not functional metrics such as self-reported frequency of toe-drag and falls were improved.

Apart from tibialis anterior single-channel NMES, one study examined single-channel stimulation to the quadriceps femoris and reported significant improvements in knee kinematics (28). This finding is at odds with a prior publication (35) and could be due to improvements in stimulation technology and timing. However, there is insufficient evidence either way, as the older publication included only four children receiving stimulation to the quadriceps, and the newer study was a case study of one participant.

#### Multi-channel NMES-Assisted Gait

Since 2010, only two case studies have reported on multi-channel NMES use in persons with CP (Table 2). Rose et al. (21) applied stimulation to the gluteals, quadriceps femoris, and gastric-soleus muscles bilaterally, in a three subject single-session case study, and Behboodi et al. (20) applied stimulation to 3–4 muscle groups bilaterally in a two subject case study.

Rose et al. (21) recruited children specifically with flexed-knee gait, defined as 20–40°C of knee flexion in stance. Stimulation was then applied to the quadriceps and gluteus muscles from initial contact through 50% of the gait cycle, and gastrocnemius-soleus complex from 15 to 60% of the gait cycle. They demonstrated improved velocity and GDI in 2 out of 3 participants with improved hip, knee and ankle joint kinematics during a single-session of NMES-assisted gait. The multi-channel NMES study published by Behboodi et al. (20) demonstrated a number of promising findings in their small case study. Their 12-week program of gait-specific NMES training did appear to be beneficial to the two individuals in the study. In all, the study demonstrated a normalization of temporal-spatial parameters with improved fitness in the participant with GMFCS III, and improved efficiency in the participant with GMFCS II, as determined by peak VO_2_ and O_2_ cost of walking, respectively.

### The Arc of Progress of Multi-channel NMES

The largest study to assess the effect of multi-channel NMES in children with CP included 17 children, 8 of whom received NMES, and was conducted in 2004 (39). This publication, and the three others studying multi-channel NMES prior to 2010 (33, 37, 40), were in the early stages of NMES research, utilizing percutaneous electrodes and custom stimulator systems. Furthermore, they were either limited in dosage (37), underpowered (40), or highly confounded by extraneous factors, such as major surgeries (39) or Botox injections (33). They were appropriate for their time, as exploratory studies, however an assessment of the efficacy of multi-channel stimulation by modern technological standards has not been attempted beyond case studies. The targeted augmentation of ankle DF in swing is an appropriate intervention for patients whose primary gait abnormality is the lack of ankle DF during swing due to DF weakness. However, it is likely to be insufficient in the majority of children with CP. It would not be expected that normalization of the kinematics of a single joint during the non-weight bearing phase would have substantive effects on proximal muscle weakness and control during stance phase. The success of achieving improvements in ankle DF by single-channel NMES is encouraging but in large part due to the muscular demand being relatively small; ankle DF in swing is non-weight bearing and acts on a small load (the foot). Similarly, forces of hip flexors in early swing and knee extensors in terminal swing are low relative to the forces necessary to achieve upright gait during stance phase. This was leveraged and partially demonstrated in the case study by Behboodi et al. in stimulating the quadriceps in terminal swing, achieving greater knee extension and a resulting increased stride length (20).

Moving forward, the results of this review suggest a possible return to the study of multi-channel stimulation in children with CP. Stimulation of the tibialis anterior and augmentation of ankle DF is likely to be a critical component of such future systems; however, it appears unlikely that it alone will resolve gait deficits seen in this population. Interestingly, gait-specific NMES applied to the tibialis anterior was shown to only improve ankle kinematics (8), while the singular recent case study on gait-specific NMES applied to the quadriceps femoris showed only improvements in knee joint kinematics (28). This taken in conjunction with the findings of earlier studies that multi-channel stimulation can have additive beneficial effects, would suggest that normalization of more involved gait deficits, would require a multi-channel gait-specific NMES acting across multiple joints. This early hypothesis is supported by the case studies published in Rose et al. (21) and Behboodi et al. (20). Additionally, a single subject case study published in 2015, assessing the effects of gait-specific multi-channel stimulation to the tibialis anterior and hamstrings in an adult with CP, demonstrated improvements in the Dynamic Gait Index, Performance Oriented Mobility Assessment, Observational Gait Scale, and Activities-specific Balance Confidence Scale scores (42). However, these are all case study examples, and the benefits of a multi-channel NMES system must be born out in a larger study.

Multi-channel gait-specific NMES development is a task not only for clinicians to carry out, but also the medical device industry and research groups. Currently, two double-channel NMES devices were found capable of initiating gait-specific stimulation in a self-regulated manner, the Bioness L300 Plus and Odstock 2 Channel Stimulator. The L300 Plus system employs a tilt-sensor to control a common fibular nerve stimulator, and a thigh mounted stimulator, for either quadricep or hamstring stimulation. Comparatively, the Odstock device uses a foot switch sensor to trigger stimulation of the gastrocnemius and tibialis anterior at mid-stance and during swing, respectively. Early stages of multi-channel device development can leverage lessons learned from wearable robotic development such as the idea that that bi-directional control of a joint is vastly more difficult than unidirectional control (43). As such, early implementations of multi-channel NMES may benefit from limiting the amount of bi-directional joint actuation.

In general, a multi-channel device is likely best utilized by applying stimulation within the normal timing of the muscle, and providing unidirectional control of the hip, knee, and ankle joints. For example, a device that provided quadriceps stimulation from terminal swing through loading response, gluteal stimulation from initial contact through loading, gastrocnemius-soleus stimulation from loading through mid-stance, and tibialis anterior stimulation in swing phase. Applying gait-specific NMES across the three major lower limb joints within periods of physiologically normal timing could provide vital sensorimotor input and biomechanical support, and lead to greater normalization of gait as compared to stimulation at a single joint.

### Limitations

The findings of this review must be taken in the context of the quality and size of the studies it is comprised of. Only eighteen relevant studies have been conducted, four of which are RCTs, all with 34 or fewer participants. Amongst the RCTs, the two larger and best conducted studies asses only TA stimulation in swing. The remaining two studies are smaller, and asses single-channel stimulation of other muscle groups, one of which being the gluteus medius which has not been repeated since. Many of the findings of this review are based on small cohort and case studies. Ultimately, at this stage in this field, this review can largely only highlight what has and what has not been observed. The authors recognize the absence of evidence in this field and have attempted to present the findings scaled with that expectation.

The recruitment and retention of study subjects from a pediatric population with cerebral palsy is difficult. Electrical stimulation, while comfortable for most, can be an uncomfortable sensation for some users. The challenges are even greater using un-refined research grade devices for many of the more ambitious multi-channel studies. The physical appearance of these devices is an additional barrier to acceptance for parents and children, and their technical requirements often constrain them to laboratory use only. It is remarkable that some of these studies maintained intervention programs and follow up out to 1 year. But it is also why many of these studies ultimately become either case studies, or single sessions. This fundamental limitation will remain until commercially available multi-channel devices are available for home and community use.

The Cochrane guidelines for risk of bias assessment for both randomized and non-randomized studies were consulted to guide an assessment of risk across all studies considered. The risk of bias across nearly all seven elements is considered high for a few key factors, mostly stemming from this being such preliminary research. The study population is a self-selected subset of patients and families both willing to try and interested in NMES technology, as well as being capable of tolerating the electrical stimulation. Although adequate random sequence generation was performed in some, recruitment is fundamentally non-random. One cohort study even defined its control group, as those who could not tolerate the electrical stimulation (33). The effects of this non-random recruitment make the smaller studies at an even higher risk for bias due to ideal patient selection. The intervention itself, is practically impossible to blind participants from, and no study blinded the personnel from. Finally, since so many studies were small cohort or case studies, there is an even higher risk of bias due to inherent selective reporting bias stemming from the results not being statistically significant in such small studies. Additionally, the reporting of small case studies could also be influenced by selective reporting of best responders. Even objective outcome data could be potentially biased by effort, since the participants are aware of the intervention. Considering the high risk of bias, the outcomes in this review are at a high risk of over-estimating the potential benefits of NMES technology.

Conducting a more focused review to address a specific clinical question was considered, however due to the under-developed nature of this field, it was determined that insufficient evidence exists to adequately answer any specific question and a broader review would be more beneficial. A systematic review at this early point, would have unduly limited the diversity of studies. It would have placed an over-emphasis on a limited few randomized control trials which have all assessed the same technology—a tibialis anterior stimulator for ankle dorsiflexion in swing. As a consequence, the results of this review do not provide the strength of evidence that a systematic review would provide.

## Conclusion

CP is a common neurological disorder presenting early in childhood with progressive musculoskeletal and functional impairments. Current pharmaceutical and surgical treatments are inadequate, with only partial benefits. Neuromuscular electrical stimulation is a well-established technology that has demonstrated recent progress due to advances in wearable electromechanical technology that can improve functional electrical stimulation treatment. The study of NMES during gait in persons with CP is still limited, however these early studies are promising in showing that NMES may offer unique benefits for gait rehabilitation. Improvements seen with NMES, such as joint kinematics and muscle volumes, still must translate to other functionally meaningful metrics such as degree of community ambulation. Further advancement of NMES technology, particularly in the arena of multi-channel devices and the targeting of major muscle groups, may yield even greater improvement in the gait of individuals with CP. Electrical stimulation is a particularly appealing technological solution as it may play a restorative or compensatory role in the four major defining musculoskeletal deficits of spastic CP, while remaining non-invasive and highly individually tunable. Overall, NMES is a well-accepted and tolerated intervention, with high reported rates of patient satisfaction and retention.

## Author Contributions

JM and JR conducted the literature search, contributed to writing and editing the manuscript, conceptualized the framework within which to conduct the review, and have reviewed and approved the final manuscript.

### Conflict of Interest Statement

The authors declare that the research was conducted in the absence of any commercial or financial relationships that could be construed as a potential conflict of interest.

## Abbreviations

TA: tibialis anterior
QF: quadriceps femoris
G: gastrocnemius
S: soleus
Glu: gluteals
GMa: gluteus maximus
GMe: gluteus medius
BF: biceps femoris
VL: vastus lateralis
VM: vastus medialis
PAM: posterior adductor magnus
DF: dorsiflexion
PF: plantarflexion
IC: initial contact
TO: toe-off
SCALE: selective control assessment of the lower extremity
PCI: physiological cost index of walking
GMFM: gross motor function measure
COPM: Canadian occupational performance measure
6MWT: 6-minute walk test
ROM: range of motion
OGS: observational gait score
GDI: gait deviation index.
